# Do not forget asthma comorbidities in pediatric severe asthma!

**DOI:** 10.3389/fped.2022.932366

**Published:** 2022-07-29

**Authors:** Lucia Ronco, Anna Folino, Manuela Goia, Benedetta Crida, Irene Esposito, Elisabetta Bignamini

**Affiliations:** ^1^Department of Pediatric Science, School of Medicine, University of Turin, Turin, Italy; ^2^Department of Surgical Science, University of Turin, Turin, Italy; ^3^Pediatric Pulmonology Unit, Regina Margherita Children Hospital, AOU Cittá Della Salute e Della Scienza, Turin, Italy

**Keywords:** asthma, severe asthma, children, comorbidities, difficult to treat asthma

## Abstract

Asthma is the most common chronic respiratory disease in childhood. The long-term goals in managing asthma aim to control symptoms and prevent exacerbations, as well as to reduce side effects of therapy and mortality disease-related. Most of patients have mild to moderate asthma and respond well to standard therapies. However, a minor proportion of children with asthma has severe disease that remains uncontrolled despite optimal adherence to prescribed therapy and treatment of contributory factors, including trigger exposures and comorbidities, which can mimic or worsen asthma and contribute to exacerbations and poor quality of life. Evaluation of comorbidities is fundamental to optimize the management of the disease in a subgroup of patients with poor responder asthma. The overall aim of this article is to describe characteristics of main pediatric severe asthma comorbidities reported in literature, giving clinicians tools to recognize and manage properly these conditions.

## Introduction

**Figure 1:**
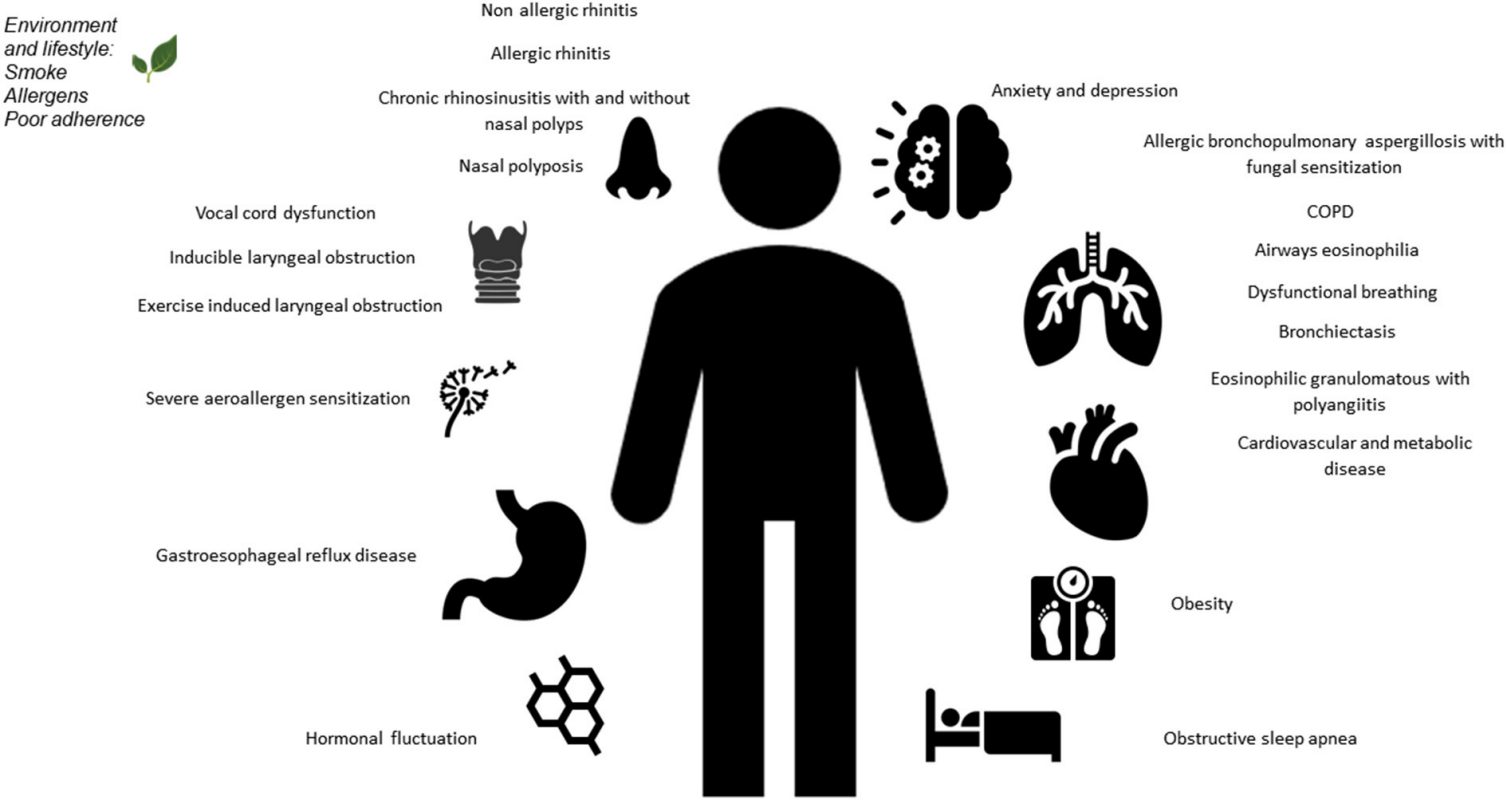
Overview of pulmonary and extrapulmonary comorbidities in severe asthma, in adults and children.

Asthma is a heterogeneous disease characterized by chronic inflammation of the airways. Clinical presentation can be various in time and intensity with asthma attacks characterized by wheezing, shortness of breath, chest tightness and cough together with a variable and reversible limitation to the expiratory flow. Most of patients have mild to moderate asthma and respond well to standard therapies (1). However, a minor proportion of children with asthma has severe disease that remains uncontrolled with continuous symptoms, frequent exacerbations and increased risk of hospitalization (2). Severe asthma is a coexistence of clinical, molecular, and cellular inflammatory characteristics and it assessed retrospectively on the level of therapy required to control symptoms and exacerbations, once the disease has been stabilized (3). Its prevalence among children with asthma is estimated up to 5% (4), with a significant socio-economic impact, requiring the consumption of 30–50% of the health resources destined for asthma in general (5). In most cases, severe asthma is related to bad adherence to therapy, incorrect use of inhalers, environmental and psychological factors and co-existing and inadequately treated comorbidities as show in Figure 1 (6). Comorbidities are diseases, disorders or medical conditions that are simultaneously present with another or others in a patient and may not have etiological association with asthma. Moreover, specific clusters of comorbidities may develop at the same time, interacting with each other and playing an aggregate effect on the individual's asthma outcomes (7). Co-occurring of different conditions in asthma is associated with more complex clinical management and worse health outcomes (8). Phenotypic differences and underlying comorbidities will impact treatment choices, therefore these conditions should be carefully assessed and properly managed to avoid inappropriate therapy and improve asthma management (9). In this review we will discuss main comorbidities associated to asthma in children, describing their possible role in severe asthma (Table 1).

**Table 1:** Main articles reporting comorbidities in pediatric severe asthma.

**Comorbidities**	**Airways eosinophilia**	**Allergic bronchopulmonary aspergillosis with fungal sensitization**	**Allergic rhinitis**	**Anxiety and depression**	**Bronchiectasis**	**Cardiovascular and metabolic disease**	**Chronic rhinosinusitis with and without nasal polyps**	**COPD**	**Dysfunctional breathing**	**Exercise induced laryngeal obstruction**	**Eosinophilic granulomatous with polyangiitis**	**Gastroesophageal reflux disease**	**Hormonal fluctuation**	**Inducible laryngeal obstruction**	**Nasal polyposis**	**Non allergic rhinitis**	**Obesity**	**Obstructive sleep apnea**	**Severe aeroallergen sensitization**	**Vocal cord dysfunction**
**Authors**																				
Jonathan M. Gaffin et al.		x	x				x		x			x	x	x			x	x		
Andrew Bush										x							x			
Paola Rogliani et al.	x	x	x		x	x	x	x	x			x				x		x		x
Celeste Porsbjerg, Andrew Menzies-Gow		x	x	x	x		x		x			x			x		x	x		x
Elizabeth Scotney, Sejal Saglani											x								x	
T. R. Tay, M. Hew				x			x		x			x				x	x	x		x
Samriti Gupta et al.												x						x		

### Obesity

Obesity is known to be an aggravating factor of many pulmonary conditions, through effects on lung mechanical function (10) and altered immunological and inflammatory state (11, 12). The interactions between asthma and obesity are varied and complex and can have causes and/or predisposing factors in common, including environmental, genetic and microbiological factors (13). Obesity is known to be significantly associated with a greater asthma severity (14) and a poorer asthma control and quality of life (15), with chronic systemic inflammation and steroid resistance as the main explanations for this correlation. Moreover dysanapsis, defined as the incongruence between the growth of the lung parenchyma and the airways caliber among overweight or obese children, has been demonstrated to worse disease severity and reduce response to treatment, as airflow obstruction is anatomical and/or developmental and thus at least partly not related to bronchospasm or airway inflammation (9). In a retrospective cohort study by Lang et al. obesity increases asthma incidence in preschoolers, school-age children and adolescent population, with the highest risk observed in the youngest subgroup, moreover depending on sex, ethnicity and allergic status (16). On the other hand, asthma itself drives an increase in the onset of obesity among schoolchildren, as the same anti-asthma drugs, in particular steroids, and poor tolerance to physical exertion can favor overweight (17). Moreover, other comorbidities like gastro esophageal reflux disease (GERD) and obstructive sleep apnea syndrome (OSAS) co-exist in obese patients, contributing to asthma poor control (18). Obesity is an easily identifiable condition, but it often leads to an incorrect diagnosis of asthma, rather than coexist as a separated comorbidity. Obese subjects in fact can be misdiagnosed as asthmatic patients, as their symptoms during exercise or bronchial challenge are equal to or higher than those experienced by asthmatic patients, without evidence of airflow obstruction or bronchial hyper responsiveness (19). This is due to high excessive ventilator response for metabolic demands and chronic mild inflammation caused by augmented proinflammatory cytokines such as leptin and reduced anti-inflammatory ones (adipokine) from the adipose tissue (12). Obesity is a modifiable risk factor for asthma and weight loss has been shown to improve control of asthma symptoms (20), so that diet, exercise and behavioral therapy should be always encouraged in obese asthmatic and non-asthmatic children and the possibility of practicing physical activity free from respiratory symptoms must always be part of the therapeutic goals (21). As suggested by Fainardi and colleagues, in a child with obese-asthma phenotype, a step-wise approach including the evaluation and management of obesity-associated comorbidities, mainly OSAS and GERD, is fundamental (22).

### Gastro esophageal reflux disease

GERD worsen asthma control, increasing odds of suffering asthma exacerbations (23) and is associated with severe asthma in a bidirectional relationship, as demonstrated by longitudinal follow-up studies (24). Its prevalence among children with asthma is estimated to be 43–87% (18). GERD is thought to enhance bronchoconstriction through vagal nerve stimulation and micro aspiration of small amount of gastric and duodenal contents that irritate and damage the airways leading to release of inflammatory cells and mediators. On the other hand, asthma pulmonary hyperinflation and increased negative pleural pressure due to bronchoconstriction increase the pressure gradient between the thorax and the abdomen, favoring reflux. Moreover, asthma therapies may worsen GERD symptoms increasing gastric acid production or lowering esophageal sphincter tone (25). Diagnosis is based on typical symptoms of regurgitation and heartburn but in some cases extra-esophageal symptoms like cough or wheezing may represent the only clinical manifestations. Lifestyle changes including postural therapies and weight loss should always be encouraged. In clinical practice, symptomatic GERD is treated with proton pump inhibitors (PPI), although data to suggest that this therapy reducing asthma severity are limited (26). A recent review investigated the pharmacological intervention to manage GERD in asthma patients, both adults and children, concluding that medical treatment of GERD has an uncertain effect on reducing exacerbations, using of rescue medications and improving respiratory function (27). PPI use has been associated with an increased risk of asthma in children and the hypothesized underlying mechanism is supposed to be the change of the microbiome in the lung and the gut, leading to a dysregulation of immunity. Therefore, these drugs should be prescribed only when clearly indicated, weighing the potential benefit against potential harm (28). Additionally, surgical treatment for GERD in people with asthma are currently under-studied and evidence is lacking, especially among children

### Obstructive sleep apnea syndrome

Episodes of complete or partial upper airway closure in OSAS are associated with blood-gas changes and altered normal sleep architecture with long-term sequelae (29). In patients with co-existing asthma, OSAS is widely known to be associated with poor asthma control and more frequent severe exacerbations (30, 31). Their co-existence is explained by common risk factors (mainly obesity and GERD) in a bidirectional relationship where an underlying pathogenetic pathway promote both upper and lower airway inflammation (31) and a neutrophilic inflammation is predominant in patients with OSAS and severe asthma (32). Moreover, repeated episodes of upper airways collapse in OSAS trigger cyclical hypoxemia and vagally-induced bronchospasm (33). The prevalence of OSAS in children with asthma is reported about 35% (34), rising up to 66% when diagnosed with polysomnography (35) and, among those with severe asthma, 63% have concomitant OSAS (36). A retrospective cohort among children hospitalized for acute asthma found that those with coexisting OSAS had higher risk of noninvasive positive pressure ventilation use and longer length of stay compared with those without OSAS (37). At the same time, the presence of asthma is associated with more severe OSAS and need for continuous positive airway pressure, but it has not been established if controlling asthma decreases severity of OSAS (38). Conversely, a retrospective case-control analysis showed that asthma might reduce the risk of OSAS, explaining this result with the point that the avoidance of airway collapsibility and reduction of systemic inflammation actually might prevent progression to OSAS (39). Being adeno tonsillectomy the first-line treatment for OSAS in children, a systematic review by Sanchez et al. summarized that surgical intervention was associated with clinically significant reductions in markers of asthma severity (40). According to this, a more recent prospective controlled study demonstrated that adeno tonsillectomy improves asthma outcomes as measured by the Childhood Asthma Control Test C-ACT, but with only minimal improvements in the asthma clinical outcomes (41). Asthmatic children with non-fully controlled asthma or frequent nocturnal symptoms or those who have risk factors for OSAS (obesity and other comorbidities) should be evaluated to rule out an underlying sleep disorder (31).

### Allergic bronchopulmonary aspergillosis and fungal sensitization

Fungal sensitization is common in asthmatic children and is associated with a worsening in asthmatic feature such as lung function, airway inflammation and bronchial reactivity (42). Airway damage in severe asthma may lead the environment be more easily colonized by fungi and fungi directly contribute to the development of severe asthma by augmenting the immunological response (43). Moreover, the alteration of mucosal immunity related to treatment with systemic corticosteroids in severe asthma patients predispose to an increased fungal load, augmenting the type 2 inflammatory response in sensitized patients (44). Allergic bronchopulmonary aspergillosis (ABPA) is caused by repeated inhalation of *Aspergillus fumigatus* spores which remain trapped in the thick sputum of patients causing a hypersensitivity reaction (45). Main clinical manifestations include frequent exacerbations, productive cough with mucus plugs, hemoptysis and constitutional symptoms as fever, weight loss and fatigue (46). Specific laboratoristic and radiological criteria are needed to diagnose ABPA among asthmatic patients and asthma is considered itself a predisposing condition to develop ABPA, besides cystic fibrosis or tuberculous disease (47, 48). Epidemiological studies to evaluate the prevalence of ABPA in children are mainly from India whereas in developed countries ABPA is considered a rare condition. The reason for this regional difference may be due to environmental factor or genetic predisposition. Kumari observed prevalence of ABPA as 11.3% in Indian children with poorly controlled asthma, without identifying any specific risk factor for ABPA (45). The goals in the treatment of ABPA aimed to reduce exacerbations, prevent deterioration of lung function and evolution to end-stage fibrotic disease (49). Systemic steroids are the most effective treatment to reduce inflammatory response, whereas efficacy of antifungal drugs to eradicate A. fumigatus is uncertain and is not currently recommended as first-line treatment with steroids in children with ABPA. Omalizumab and mepolizumab are monoclonal antibody recently proposed as an alternative treatment in ABPA and asthma (50).

### Allergic rhinitis

Allergic rhinitis (AR) is considered a major risk factor for asthma onset and uncontrolled or moderate-to-severe AR can significantly affect asthma control, being associated with more frequent wheeze attacks (51). Chronic disease with inflammation of the nasal mucosa and nasal airway hyper reactivity in AR is caused by exposure to inhaled allergens in a sensitized patient (52). Interactions between the upper and lower respiratory tracts are well known as they share anatomical, functional, pathogenic and immunological patterns (53), so that allergic airway disease represents a continuum of a single inflammatory process (54). Moreover, the impaired function of the upper airways in AR leads to reduction of filtering, warming and humidifying air before it reaches the lower airways, causing inhalation of cold dry air and greater delivery of allergens (55). AR prevalence estimated is between 10 and 30% of children and adults (56), whereas ~60–80% of children with asthma have AR (4), considering asthma itself as a major risk factor for the onset of AR (54, 57) and, on the other hand, severity of AR has shown to be associated with poor degree of asthma control (58). The diagnosis of AR is based on clinical presentation with itching, nasal discharge, sneezing and nasal airway obstruction. Skin prick test, with serum specific Immunoglobulin E and allergen provocation tests are used as a second-line tests when other investigations are inconclusive (59). Current treatment for children with asthma and AR include allergen avoidance whenever possible and standard pharmacotherapy to manage and reduce symptoms, mainly using oral and intranasal H1-antihistamines, intranasal corticosteroids and leukotriene receptor antagonists (60). Allergen-specific immunotherapy, either in the subcutaneous or sublingual form, has a immune modulator effects by augmenting the production of IgG in the serum and IgA in nasal secretions and lowering specific IgE (61). Despite having been shown to be safe in children as young as 3 years of age, in clinical practice it remains secondary to symptomatic therapies probably due to its elevated costs and lack of awareness of its clinical efficacy in children with asthma, which remains controversial (62).

### Chronic rhinosinusitis

Chronic rhinosinusitis (CRS) is an inflammatory condition in the nose and paranasal sinuses (63). It has been demonstrated to be associated with impaired asthma control and increased exacerbation frequency (64), in a common ground due to a systemic cyclic inflammatory response (65), which is not only by contiguity between upper and lower airways, but it is the result of a complex interplay among several immunological mechanisms both inside and outside the respiratory system (66). Moreover, chronic inflammatory process leads to a remodeling process of sinonasal tissues, with epithelial edema, basal membrane thickening and polyps formation, similarly to lower airways remodeling occurring in asthmatic patients (67). Diagnosis is made clinically, recurring to nasal endoscopy to identify purulent drainage and the presence of polyps protruding in the nasal cavities, distinguishing in that way CRS with (CRSwNP) or without (CRSsNP) nasal polyps (63). Epidemiological association between CRS and asthma is quite clear. In a study of Marseglia et al. asthmatic children were investigated by nasal endoscopy and occult sinus involvement was demonstrated in 7.5% of them, who resulted to have poorly controlled asthma, suggesting that it is reasonable that children and adolescents affected by poorly controlled asthma should be investigated for occult or manifest CRS (68). More recently researchers followed adults and pediatric patients for 5 years after the diagnosis of CRS and found that they were at increased risk to develop respiratory diseases, including asthma (69). Treatment is aimed at reducing airway inflammation with saline washes and sprays, intranasal and systemic corticosteroids, antibiotics and antileukotriene agents (70). In patients in whom these medical interventions do not result in sufficient improvement in symptoms, surgical treatment (endoscopic sinus surgery or polypectomy) could take place. Biologic agent as dupilumab are not validated to treat CRS in pediatric population, being approved in adults and adolescents with moderate-to-severe asthma with an eosinophilic phenotype or with oral corticosteroid-dependent asthma, and in adults only for severe CRSwNP (71).

### Dysfunctional breathing

Dysfunctional breathing (DB) is as an alteration in the normal biomechanical pattern of breathing that result in intermittent dyspnea, wheezing, cough and upper chest pain and other non-respiratory symptoms (4). DB is associated with asthma morbidity through a number of potential mechanisms and a complex interrelationship including anxiety, psychological disorders and emotional distress (72). As symptoms of DB may mimic or be mistaken for those of asthma, identifying DB as a comorbidity complicating asthma attack or severe asthma can be challenging as there is considerable overlap in these conditions. In addition, when hyperventilation is documented, there might be symptoms that overlap also with anxiety, for example dizziness, palpitation, paresthesia, lack of concentration and fatigue (8, 73). An association of DB and poor asthma control was strong and well documented in a study population of 760 Italian adolescents, where DB were ten times more common in subjects with asthma (25%) than in those without asthma (2,5%) (74). Hepworth et al. reported a higher prevalence of DB symptoms (35%) in a cohort of children, probably due to the baseline suspicion of DB and the consequent referral to physiotherapist intervention (75). Directly observing breathing pattern by a specialist respiratory physiotherapist is useful to detect DB, as it provides a semi-objective tool to characterize DB in treatment-refractory asthma (76). Other objective assessments have been tested and validated, including the cardiopulmonary exercise testing and hyperventilation provocation test (77). An early referral to an experienced respiratory physiotherapist, a specialist in speech and language therapy or a psychologist might help to manage DB and to improve asthma symptom in children (73). A review of randomized controlled trials evaluated the effects of breathing exercises in children with asthma but did not draw reliable conclusions, due to the unclear risk of bias and the low quality of the evidence (78), despite breathing exercise intervention improved significantly asthma symptoms (75).

### Vocal cord dysfunction

Vocal cord dysfunction (VCD) is an involuntary adduction of vocal cords during inspiration that can be misdiagnosed as asthma or can amplify symptoms of asthma, with poor or no response to asthma medicaments and unavoidable persistent poor asthma control (79). Symptoms can be very similar to those of asthma and vary from mild dyspnea to acute breathlessness, whereas inspiratory stridor (often mistaken for wheezing) is the hallmark presentation of VCD (80, 81). The cause of VCD is unknown, but functional component due to psychological stresses is thought to be the most involved. Also exercise, upper respiratory tract infections and local irritation (e.g., smoke, chemical irritant, reflux) that lead to increased laryngeal sensitivity can be responsible for VCD (81). Among pediatric patients, VCD prevalence is not well established as it can mimic asthma (82), or it can coexist with asthma (83) masquerading as a difficult to treat asthma. It is largely known to be more frequent in female patients and in elite or intense young athletes (79, 81, 84). The gold standard for diagnosing VCD is fiberoptic laryngoscopy, which ideally should be performed while the patient is symptomatic or under circumstances that elicit VCD symptoms (81), as exercise, methacholine or irritant substances. In patients with exertional dyspnea, continuous laryngoscopy exercise-test (CLE) during physical exercise reveals VCD occurring and peaking during exercise, whereas in case of asthma, it usually peaks 5–20 min after the end of exercise (79). As laryngeal dysfunction responds to speech pathology intervention, psychotherapy coupled with breathing techniques are considered the milestone treatment of VCD (9). Pharmacological therapies are used to treat co-existing or triggering comorbidities of VCD, as rhinitis or GERD. Ipratropium or injection with Clostridium botulinum toxin (off-label in the pediatric population) into laryngeal muscles can also be considered (81).

## Conclusions

Severe asthma in children remains a clinical challenge. Comorbid conditions may complicate asthma management or can lead to misdiagnosis of asthma, with consequent undertreatment or overtreatment. Identifying asthma comorbidities is essential to better asthma and severe asthma management and to improve symptom control and patients' quality of life. Multiple comorbidities can coexist in the same patient, as GERD and obesity, moreover some comorbid conditions may not have a clear etiological association with asthma, being a coincidental finding, as ABPA or VCD. All possible risk factors for comorbidities need to be investigated to ensure the maximal effort to get symptoms control. An appropriate multidisciplinary assessment and a stratified diagnostic approach are mandatory for the best management and treatment of comorbidities.

## Author contributions

LR and AF: critical revision of the article. BC, MG, and IE: drafting the article. EB: conception or design of the work. All authors contributed to the article and approved the submitted version.

## Conflict of interest

The authors declare that the research was conducted in the absence of any commercial or financial relationships that could be construed as a potential conflict of interest.

## Publisher's note

All claims expressed in this article are solely those of the authors and do not necessarily represent those of their affiliated organizations, or those of the publisher, the editors and the reviewers. Any product that may be evaluated in this article, or claim that may be made by its manufacturer, is not guaranteed or endorsed by the publisher.
